# Supplementary material for: The Heart Health Yarning Tool: Co‐Designing a Shared Decision‐Making Tool With Aboriginal and Torres Strait Islander People for Cardiovascular Disease Prevention and Risk Management
Source: Health Expect. 2025 Aug 17;28(4):e70387. doi: 10.1111/hex.70387 (PMC12358686; doi:10.1111/hex.70387)
Supplement: Supplementary file 2 [file HEX-28-e70387-s004.docx]

**Supplementary file 2**

**Guidance for Reporting Involvement of Patients and the Public 2 short form (1)**

| **Section and topic** | **Item** | **Reported on page No. / extended response** |
| --- | --- | --- |
| 1. **Aim** | Report the aim of PPI in the study | P8-9 (Study design). We also aimed to ensure appropriate and culturally sensitive conduct of the study/research team by consulting with our Aboriginal and Torres Strait Islander Reference Group on study design, dissemination of results and outcomes, appropriate next steps. |
| 1. **Methods** | Provide a clear description of the methods used for PPI in the study | P9 (Study design), P10-11 (Participants, Data collection) p13 (Patient and public involvement) |
| 1. **Study results** | Outcomes—Report the results of PPI in the study, including both positive and negative outcomes | P15-17 (Results – Phase 1), p19 (Results – Phase 3). |
| 1. **Discussion and conclusions** | Outcomes—Comment on the extent to which PPI influenced the study overall. Describe positive and negative effects | P20 (Discussion). As a result of engaging community members, together with health professional stakeholders, we were able to develop a co-designed tool to support health professionals make shared decisions about heart health with Aboriginal and Torres Strait Islander people. Community members who participated in yarning workshops and interviews also reported that they gained an increased understanding of heart health issues |
| 1. **Reflections/critical perspective** | Comment critically on the study, reflecting on the things that went well and those that did not, so others can learn from this experience | P23-24 (Strengths and limitations). |

PPI=patient and public involvement

1. Staniszewska S, Brett J, Simera I, Seers K, Mockford C, Goodlad S et al. GRIPP2 reporting checklists: tools to improve reporting of patient and public involvement in research BMJ 2017; 358 :j3453 doi:10.1136/bmj.j3453
