# Supplementary material for: The Heart Health Yarning Tool: Co‐Designing a Shared Decision‐Making Tool With Aboriginal and Torres Strait Islander People for Cardiovascular Disease Prevention and Risk Management
Source: Health Expect. 2025 Aug 17;28(4):e70387. doi: 10.1111/hex.70387 (PMC12358686; doi:10.1111/hex.70387)
Supplement: Supplementary file 3 [file HEX-28-e70387-s003.docx]

Supplementary file 3:

**Table 1 (extended version): Examples of interview data mapped to Finding Your Way (FYW) shared decision-making processes**

| **FYW step/process** | **FYW description** | **Example of mapped data / Illustrative quote** | **Heart Health Yarning Tool content** |
| --- | --- | --- | --- |
| Options | Yarn about the options and the benefits and risks of treatment. Ask questions, share knowledge and feelings about the treatment options | *The options gives us control, it gives us that power over our own – making decisions about our own treatment and what we want and our own needs, and I really like that.* | Which options do you want to consider to improve your heart health? |
| Support | Yarn about what is happening today, including social, emotional and wellbeing needs and supports | *We used to go to walking groups, and they said you might be interested in endocrinologist via telehealth, and it’s been beneficial. It’s something that I would not have considered, and I found it a very beneficial referral.* | You have more in your life than just your heart health needs, talk about what else is important to your social and emotional wellbeing.  You can yarn to family, friends, health professionals and other people in your community about what you might need. Support can come from a team of health professionals, not just your health worker or doctor.  It’s important for the people who are looking after you to know what works well for you, to support you in the best way for you. |
| Family or friends | Yarn about family and mob. If needed, bring others into the yarn for support. | *If you’re going to give positive feedback to family about your experience, they’re going to feel more entrusted to go as well and see about their heart. Yeah, so I think it’s a really good tool and I think it just outlines the importance of yarning.* | Talking about your family and friends, your country and their experiences of heart and vascular issues can be helpful.  Yarn with your family and friends about your options. It is okay to make these decisions together with family.  Making decision with others is ok. You can bring family or friends to the doctor to help you make decisions.  You might all make some changes together to improve your health e.g. you might all go for a walk together every day.  Your family may have had to find solutions to improve their health in the past which may work for you too. They may have some tips they can share with you. |
| Ways of knowing, being and doing | Yarn about ways of knowing, being and doing to inform health decisions based on a person’s values and beliefs | *When I went over to one of our clinics, I really felt heard, I really felt respected, it wasn’t rushed and they actually sat down and treated you like a human being. They didn’t lecture you. They actually talked about looking at our strengths as to how we can improve our health and focusing on what was wrong type thing. So, you felt like a human being.* | You are the expert about your story and your body. Nobody knows your journey better than you do, and everyone’s journey is different. Here are some questions you can yarn about with your health professional to help them understand what’s important to you. |
| Weigh up the odds | Yarn about the possible benefits and risks. Compare options and weigh up the odds individuals and for family and mob | *You can ask questions. What are my options? What are the benefits and harms? How likely are they to happen to me?* | There are different ways that you can improve your heart health. There will be good and bad things about each of these options.  Think about which heart health options are best for you, your personal circumstances and what support you have from family, friends and your community. |
| Take a break | Yarn about taking a break (if needed) and making the decision later. Come back and yarn on another day | *You’re not going to get all this done in one session. So, being able to have that follow-up, being… having that ongoing care, having the ongoing talking about, sharing our stories – having something you can take away and being followed up is very important.* | You might need more time to think about the options, or yarn with family and friends. It’s ok to come back another time if you’re not ready to make a decision yet.  If you don’t feel like you’re in a safe place to yarn with someone you trust, you can talk to a different health professional. You can ask about seeing an Aboriginal and Torres Strait Islander Health Worker or Practitioner if you don’t feel comfortable with your GP or nurse. You can ask to see a male or female health professional to help you feel more comfortable. |
| Next steps | Yarn about the next steps, including how and what to do next and what might get in the way. Follow up later | *This is what stuck with me, that he was naturally curious, and he actually asked me “What are the challenges, to you, getting a screen right here, right now?” Instead of giving me a lecture what he was saying was, “What can I do to help you to consider a screen?”* | When you are trying to make changes for your health some things work well and some things take more time to put into place.  Some things you try may not work at all for you, at first – talk to your healthcare team and family about how they can support you to help you find what is best for you.  Checking in is important and bring the people who support you on your health journey. |
| Decisions | Yarn to bring it all together and either decide to act now if ready, or wait | *I’d like to hear from a doctor’s point of view obviously because that’s their profession, but I also want them to hear what I’m saying and be able to answer it in a civilian way so it’s understandable… not just chucking big words around… make you understand so you don’t go home with a worried mind.* | Think about the lifestyle or medication options you want to consider. Yarn with your health professional about which ones might be best for you right now. |
